# The innovation and practice of the “hand as foot” teaching method in the teaching of Parkinson’s disease

**DOI:** 10.3389/fnagi.2025.1519067

**Published:** 2025-04-15

**Authors:** Jingjing Yang, Dandan Yang, Jie Yu, Jiahui Liu

**Affiliations:** ^1^Department of Neurology, Baotou Central Hospital, Affiliated Baotou Central Hospital of Inner Mongolia Medical University, Baotou, Inner Mongolia, China; ^2^Department of Neurology, Baotou Central Hospital, Baotou, Inner Mongolia, China; ^3^School of Traditional Mongolian Medicine, Inner Mongolia Medical University, Hohhot, Inner Mongolia, China; ^4^Baotou Central Hospital, Baotou, Inner Mongolia, China

**Keywords:** “hand as foot” teaching method, Parkinson’s disease, medical education, innovation, dopaminergic neurons

## Abstract

**Background:**

Based on years of clinical teaching experience, the orthopedic team pioneered the “hand as foot” teaching method, initially applied in orthopedic teaching. The advantage of “hand as foot” teaching lies in the high similarity of anatomical structures between upper and lower limbs, joints, nerves, blood vessels, etc., This method allows using feet to explain knowledge instead of hands. It has since expanded to internal medicine, surgery, obstetrics, and gynecology, significantly impacting clinical teaching and medical education across departments. Utilizing simple gestures or body language to grasp clinical knowledge intuitively and repeatedly has proven effective. Over years of theoretical teaching, understanding brain anatomy, particularly the anatomy and communication of Parkinson’s disease (PD), has posed challenges. Recently, after consulting relevant literature and articles on the application of the “hand as foot” teaching method in clinical teaching, I have been inspired. The invisibility and inaccessibility of brain anatomy make it challenging for students to comprehend and retain knowledge of basal ganglia anatomy. Through numerous clinical teaching experiences, we have discovered that using “hand as foot” is well-suited to address these teaching difficulties. Although there are many existing articles on manualized teaching methods in various disciplines, there is a gap in research on the impact of “hand as foot” method on students’ academic performance. The aim of this study was to investigate the impact of the “hand as foot” teaching method on knowledge related to PD.

**Methods:**

A self-designed questionnaire was used to validate the effectiveness of the “Hand and Foot” teaching method for teaching key points to undergraduate clinical medical students in Inner Mongolia Medical University.

**Results:**

There were a total of 81 participants of which 41 were in class 1 (students using “hand as foot” teaching method) and 40 were in class 2 (students using “hand as foot” teaching method), 75.61% of the students in class 1 found the “hand as foot” method useful and only 19.51% found the “hand as foot” method average. Of the students who were taught “hand as foot,” 80.49% correctly answered the option “Main pathological changes in PD.” A total of 70.73% correctly answered the option “Main components of the basal ganglia.” A total of 82.93% correctly answered the option “Typical symptoms of PD.” A total of 51.22% correctly answered the question related to the swallow tail sign. The percentage of correct answers was much higher than that of the students in the class 2. From the questionnaire survey of medical students’ knowledge of PD, we can draw several important conclusions. A total of 75.61% of the students in class 1 who had used the “hand as foot” method teaching in PD found the method helpful. The results with statistical difference (*P* < 0.05) showed that the “hand as foot” teaching method directly affected the students’ knowledge about PD.

**Conclusion:**

The “hand as foot” teaching method is generally well-received by medical students. The teacher-student interaction is good, and the understanding and memorization of difficult knowledge points are effective. It also helps visualize knowledge and strengthens students’ understanding and memory of complex concepts. This teaching approach has garnered positive feedback from both teachers and students. As basic education evolves, classroom teaching methods are continuously being reformed and advanced. Therefore, it is crucial to establish engaging teaching methods in the classroom. The analogy teaching method effectively stimulates students’ interest in learning and encourages their self-directed learning. In comparison to the indoctrination teaching model, which focuses on rote memorization, this teaching method is more likely to spark students’ interest in knowledge and boost their enthusiasm for learning. The results showed that the “hand as foot” teaching method significantly improved the correctness of the knowledge related to PD. In general, the use of “hand as foot” in the classroom can enliven the educational environment and enhance students’ understanding and retention of abstract concepts. It aids in consolidation and review after class, simplifying complex questions into an intuitive and concrete form, which is highly beneficial for clinical teaching.

## 1 Medical education, innovation

Although the introduction of multimedia in recent years has greatly enriched teaching methods, there are still many deficiencies in the use of multimedia, pictures, and models as teaching aids. Multimedia presentations, especially in PPT, mainly display two-dimensional images, providing students with a flat representation. However, real brain anatomy is a three-dimensional structure, which means that multimedia cannot provide students with a realistic experience. Additionally, due to limited space, students are unable to closely observe teaching aid demonstrations. Moreover, teaching with models is confined to the classroom, preventing students from reviewing the material after class, thus hindering the enhancement of teaching quality. China, as a developing country, particularly in the central and western regions, faces challenges in disseminating advanced technologies. The underdeveloped economy and relatively backward medical education system contribute to the limitations in teaching methods. Insufficient investment in teaching resources and the complexity of utilizing advanced teaching techniques further compound the issue. These factors make it challenging to effectively present teaching content engagingly, hindering students’ comprehension and retention of key concepts. Therefore, clarifying and simplifying complex topics for students to grasp and remember more easily is a significant challenge in theoretical teaching. Recognizing the teaching characteristics and clinical practices of the PD course, the orthopedic teaching team at the Affiliated Hospital of Inner Mongolia Medical University pioneered the “hand as foot” teaching method. This method was implemented in clinical teaching and yielded positive outcomes.

In recent years, we have read some articles in your journal about the “hand as foot” teaching method, which was first developed and implemented in orthopedic teaching. This method has been successfully applied across various subjects, yielding positive teaching outcomes. For example, application of the “hand as foot” analogy method in teaching the anatomy of palatine bones, the pterygoid plexus, superior mesenteric artery syndrome and mandibular condyle anatomy, etc., The “hand as foot” teaching method effectively illustrates numerous human anatomical structures using both hands, making it a dynamic teaching tool for medical students. Research has shown that many medical students encounter difficulties comprehending and retaining the intricate anatomy of the brain during clinical instruction. With the rapid development of medicine, the requirements for the quality and ability of medical students are also gradually improving. It is of great significance to seek a teaching method that can enhance students’ clinical and critical thinking abilities, as well as improve the overall quality of students and the teaching proficiency of clinical instructors. Mastering the anatomy of the brain is fundamental to students’ comprehension of brain disorders. In the process of clinical teaching, the focus and challenge lie in enabling students to better understand and retain the anatomy of the brain (Liu et al., 2021; Liu et al., 2022). Recently, we came across articles in journals discussing the “hand as foot” teaching method, which greatly inspired us. Subsequently, we implemented this method in clinical teaching and achieved satisfactory results. Therefore, this article will illustrate the anatomy of the brain using the “hand as foot” teaching method to make it more engaging. This “hand as foot” teaching method can assist students in grasping the anatomy of the brain more directly, laying a solid groundwork for future clinical diagnosis and imaging interpretation.

## 2 The innovative source of the “hand as foot” teaching method

The innovation of the “hand as foot” teaching method is rooted in the theory of evolution. This method leverages the high similarity of anatomical structures, such as bones, nerves, and blood vessels, between the upper and lower limbs. By using the upper limbs to demonstrate brain anatomy, students can develop a three-dimensional understanding of anatomy. Additionally, this approach encourages students to explore the “similarities and differences” between upper limb diseases and PD. By comparing and summarizing this knowledge, students can deepen their understanding and enhance memory retention. Teachers can streamline the teaching content on PD, reducing teaching time and enhancing teaching efficiency. The “hand as foot” teaching method, in conjunction with PPT presentations and model teaching aids, is employed to complement the neurology course (He et al., 2021).

## 3 Application of “hand as foot” teaching method in the teaching of Parkinson

### 3.1 Simulation demonstration of corpus striatum

**FIGURE 1:**
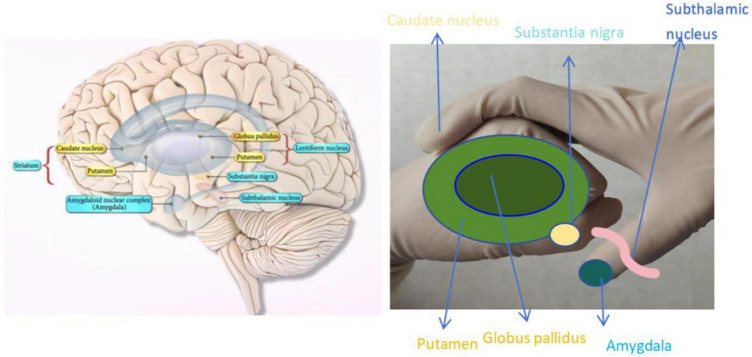
Gestures of corpus striatum.

The striatum comprises the lenticular nucleus and the caudate nucleus, which collaborate to engage in a complex array of cognitive and affective activities that regulate the learning and execution of adaptable behaviors (Fang and Creed, 2024). Lesions affecting the striatum and substantia nigra can manifest as hypertonia and hypokinesia syndrome, characterized by increased muscle tone, reduced movements, and resting tremors. This syndrome is more prevalent in PD and Parkinsonism. Neostriatal lesions can lead to hypotonia-hyperkinetic syndrome, primarily causing chorea, athetosis, and hemi-throwing movements. Lesions in the putamen may result in chorea-like movements, characterized by non-repetitive, irregular, and purposeless rapid movements; Caudate nucleus lesions may cause athetosis, characterized by slow, worm-like movements of the fingers and toes; Lesions in the thalamic nucleus may lead to hemi-throwing movements, characterized by large, forceful movements of one limb. These syndromes can be observed in conditions such as rheumatic chorea, hereditary chorea, Wilson’s disease, etc., In class, we simulated the structure of the basal ganglia and incorporated it into our theory lesson. In Figure 1, we used the right-hand bent posture to symbolize the caudate nucleus and the thumbnail bed to symbolize the amygdala. The dark green circle around the index finger and thumb of the left hand represented the globus pallidus, while the light green circle represented the shell. Yellow circles were utilized to indicate the substantia nigra, and pink lines were used to represent thalamic nuclei. Through simple gestures, the anatomical structure of the corpus striatum can be clearly expressed, and students and young doctors can easily remember every part that is easy to be confused, and improve the learning efficiency (Li et al., 2022).

### 3.2 Simulation demonstration of swallow-tail sign

**FIGURE 2:**
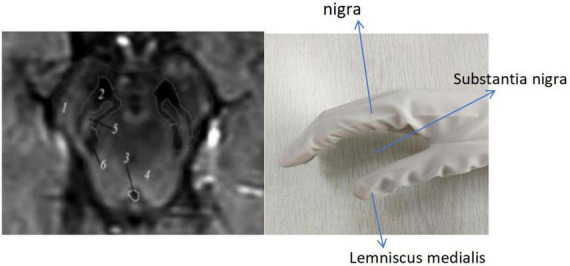
Gestures of Swallow-tail sign.

Parkinson’s disease pathology is characterized by progressive loss of dopaminergic neurons in the substantia nigra. Previous studies have identified five bodies in the substantia nigra, with substantia nigra-1 being the main structure affecting the pathological changes in PD. A study discovered that iron deposition in the substantia nigra of PD patients was significantly higher than in normal individuals, resulting in a round-like hypointense area on imaging. In normal individuals, the dense part of the substantia nigra contains a large amount of melanin, appearing as a homogeneous hyposignal area on imaging, while the substantia nigra-1 body presents as a comma-shaped, strip-shaped hyperintense area. The substantia nigra-1 body, along with the dorsal substantia nigra and ventral medial thalamus, form a “dovetail” shape (Schwarz et al., 2014). As PD progresses, a substantial amount of iron accumulates in the substantia nigra-1, reducing its signaling and causing the disappearance of the characteristic “dovetail” sign (Blazejewska et al., 2013). The diagnosis of this disease primarily relies on the patient’s medical history, clinical symptoms, and response to dopaminergic drugs, lacking an objective basis. Fortunately, imaging offers a diagnostic method, where the “swallowtail sign” can be observed in the substantia nigra SWI sequence of the midbrain in healthy individuals but not in Parkinson’s patients. The “swallowtail sign” is highly accurate in distinguishing between Parkinson’s and non-Parkinson’s patients. In Figure 2, the analogy used involves the curvature of the right hand representing the dovetail, the proximal index finger symbolizing the substantia nigra, the nail bed indicating the medial colliculus, and the gap between the thumb and index finger representing the substantia nigra nucleus. In summary, “hand as foot” teaching method can be used to visualize Swallow-tail sign that cannot be seen visually. The advancement of “hand as foot” teaching method in the field of teaching has also contributed to the progress of internal medicine and surgery (Han et al., 2021).

### 3.3 Simulation demonstration of corticosteroid thalamic-cortical (CSTC) circuit

**FIGURE 3:**
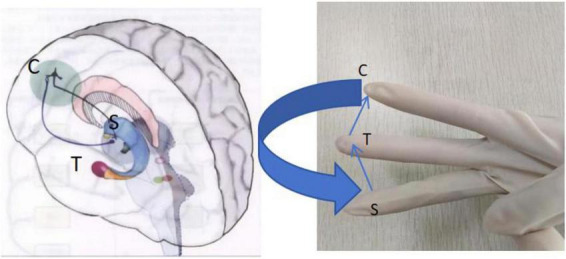
Gestures of corticosteroid thalamic-cortical (CSTC) circuit.

Degenerative changes in dopaminergic neurons are a central pathologic mechanism in PD. Through gestural simulation, students can better understand the function of dopaminergic neurons and their changes in PD. The fingers of both hands are used to represent the projection pathways of dopaminergic neurons. The projection of dopaminergic neurons from the substantia nigra to the striatum is simulated by stretching and flexing the fingers. Simulates symptoms of PD such as tremor, tonus, and bradykinesia in dopamine deficiency by bending and stretching the fingers. By hyperextension of the fingers, it simulates the hypermotility that results in dopamine excess, such as tics and dyskinesia. Through these descriptions thereby facilitating students’ understanding of the pathomechanisms of PD. The dopaminergic system is an essential rewards system in the human body, playing a crucial role in regulating emotions, learning, cognition, rewards, social behaviors, and other behaviors (Grace, 2016). Dopaminergic neurons are primarily located in the ventral tegmental area of the midbrain, the substantia nigra dense area of the midbrain, the hypothalamus, and its peri ventricles. Dopaminergic neurons can be categorized into multiple subpopulations, and different subsets of dopaminergic can project to various brain regions through five neural projection pathways: the substantia nigra-striatal dopaminergic pathway; Midbrain-limbic dopaminergic pathway; midbrain-cortical dopaminergic pathway; Nodules – infundibular dopaminergic pathway; and thalamic dopaminergic pathway. Recent advancements in neuroscience have introduced newer, more sophisticated perspectives on these pathways in schizophrenia. Here, we focus on the classic pathway: the substantia-striatal dopaminergic pathway. The substantia nigra-striatal dopamine pathway extends from the substantia nigra to the basal ganglia or striatum. It is part of the extrapyramidal nervous system and plays a crucial role in regulating movement. In untreated schizophrenia, activation of this pathway is considered a “normal” striatum central to motor control. Consequently, the substantia nigra-striatal dopaminergic pathway has traditionally been viewed as part of the extrapyramidal nervous system and interacts with the CSTC circuit or thalamic complex in the circuit. In simple terms, the nigrostriatal dopamine pathway is thought to control movement by connecting to the thalamus and cortex, forming the CSTC circuit. In Figure 3, we use three fingers to represent the CSTC circuit: the ring finger for the cortex, the middle finger for the thalamus, and the index finger for the striatum. In conclusion, neural projection pathways are intricate and complex, and it is difficult for clinicians to master them skillfully, we adopt a new teaching method of “hand as foot” to represent the CSTC circuit, which can deepen clinicians’ understanding of its anatomical structure and help them in clinical work by turning the abstraction of CSTC circuit into concrete. The “hand as foot” teaching method makes the teaching in the clinic visual and concrete, which is a very excellent teaching method (Zhang et al., 2022).

## 4 The advantages of analogical induction in the “hand as foot” teaching method

In order to show the advantages of the “hand as foot” teaching method more comprehensively, this study compares it with traditional teaching methods (e.g., PPT-based lectures) in the following aspects. In the following aspects, “hand as foot” emphasizes physical participation and interaction, and students need to do hands-on work, physical expression and teamwork. The learning process is more active and positive, with a higher level of participation. Traditional teaching is based on the teacher’s lecture, and students receive information passively, which is easy to produce distraction and fatigue, and the degree of participation is relatively low. “Hand as foot” teaching method in knowledge comprehension and memorization, through multi-sensory experience and practical operation, students are able to understand abstract concepts more profoundly and relate them to concrete experience, and memorize them more firmly. Traditional teaching relies mainly on auditory and visual information, and students’ understanding of knowledge may remain superficial, with relatively weak memorization effects. In Skill development aspect, the “hand as foot” method focuses on practice and application, and students can exercise their hands-on ability, expression ability, teamwork ability, etc., which is more conducive to the mastery and transfer of skills in the learning process. Traditional teaching is mainly based on theoretical knowledge transfer, students lack of practical opportunities, skills development is relatively weak. In terms of classroom atmosphere, the use of “hand as foot” can make the classroom atmosphere more active and relaxed, with frequent interaction between students, and the learning process is full of fun (Chang et al., 2021). In traditional teaching, the classroom atmosphere is relatively serious, with less interaction between students, and the learning process may be boring. In terms of applicability, the use of “hand as foot” requires hands-on, practical subjects, such as science, art, physical education and so on. Traditional teaching is suitable for subjects that are highly theoretical and require systematic explanation, such as history and philosophy. For students, the “hand as foot” teaching method can not only help teachers sort out and optimize the integration of knowledge points but also shorten the teaching time. Through this method, students’ self-directed learning ability is exercised, and students are guided to lead by example. In the process of learning, students not only master knowledge but, more importantly, master a learning method. Compared with traditional methods, students can remember more effectively and improve the teaching effect (Ni and Sun, 2023). Therefore, while learning through the “hand as foot” teaching method, students can also innovate and enrich the gesture teaching method based on their understanding and thinking, and summarize more knowledge points through analogy. This greatly enriches the “hand as foot” teaching method, promotes the development of medical teaching, and forms a virtuous dynamic circle of sustainable development. In clinical practice, we often encounter some difficulties in clearly expressing anatomical structures and pathophysiological processes through language, which brings obstacles to medical teaching and doctor-patient communication. Fortunately, now we can simplify and vividly describe such situations through this handy image learning method so that the communication between teachers and students, and doctors and patients, is more efficient (Liang et al., 2022).

## 5 Limitations of the “hand as foot” teaching method

Although the “hand as foot” teaching method has many advantages, there are also some potential limitations that need to be further explored and addressed. For example, not all students are suitable for or enjoy the “hand as foot” teaching method. Some students may have difficulty in adapting to this highly interactive and physically engaging teaching method due to their introverted personality, poor body coordination, learning style preference, etc., Teachers should flexibly adjust their teaching strategies according to students’ individual differences, for example, by providing different choices of activities and taking into account students’ characteristics when working in groups. Or for students who are not comfortable with the method, they can start with simple activities and gradually increase the difficulty and interactivity, giving them sufficient time to adapt. Other limitations include the fact that some of the “hands-on” activities may require students to have a certain level of subject knowledge, e.g., basic anatomy, physics, etc., otherwise it is difficult for them to understand the concepts and principles behind the activities. The solution to the above problem is that teachers should explain and prepare the necessary knowledge to help students build up the relevant knowledge base before carrying out the “hand as foot” teaching activities. Alternatively, the design of the activity could be simplified to make it less difficult to understand, for example, by using more visual aids and providing more detailed step-by-step instructions. Other limitations include the fact that the “hand as foot” teaching method usually requires more teaching resources, such as activity space, teaching aids and materials, which may be difficult to meet in some schools. Simple and easy-to-implement hands-only activities can be designed by making full use of existing resources, such as classroom space and daily necessities. Alternatively, a resource-sharing mechanism can be set up among schools to address these limitations. While there are many advantages to the “hand as foot” teaching method, statistical analysis is needed to further verify its feasibility.

## 6 Concluding remarks

Through the “hand as foot” teaching method, teachers can integrate “teaching” and “learning,” students to engage both their “hands” and “multimedia teaching materials” with “hand” teaching aids in post-class consolidation and review. It simplifies complex questions into an intuitive and concrete form, which is highly advantageous for clinical teaching, enhancing teaching effectiveness. The “hand as foot” teaching method encourages students to participate actively, enhancing their enthusiasm for learning (Huang and Xie, 2024; Li and Fan, 2024). It can complement the traditional PPT teaching method. The teaching method of “hand as foot” has been widely applied in clinical teaching (Yang and Gao, 2023; Yang and Ning, 2023), highly praised by students for its ease of understanding, and is worth promoting (Su et al., 2022).

## Data Availability

The datasets presented in this study can be found in online repositories. The names of the repository/repositories and accession number(s) can be found in the article/supplementary material.
